# Heat shock increases the anti-inflammatory and anti-obesity activity of soybean by increasing polyphenol, antioxidant and aglycon form isoflavones

**DOI:** 10.1016/j.heliyon.2023.e21944

**Published:** 2023-11-08

**Authors:** Soyema Khatun, Taewan Kim, Md. Mahi Imam Mollah

**Affiliations:** a Crop Physiology Division, Bangladesh Institute of Nuclear Agriculture, Mymensingh-2022, Bangladesh; b Department of Food Science and Biotechnology, Andong National University, Andong-1375, Republic of Korea; c Department of Entomology, Patuakhali Science and Technology University, Patuakhali-8602, Bangladesh

**Keywords:** Soybean, Heat shock, Isoflavone, Polyphenol, Antioxidant, Anti-inflammatory, Anti-obesity

## Abstract

We know that heat shock can activate the functional components in soybeans, but we don't know the type, level, and duration of heat shock for maximum activation. To address this, the present study investigated the changes in functional components like polyphenols, antioxidants, and isoflavones in soybeans at various temperature levels and durations with their respective functionality or health benefits. For this, treated seed samples were extracted with 70 % ethanol. Heat shock at 60 °C for 2 h increased polyphenol content (60.67 % of control) and antioxidant activity for both ABTS (41.14 % of control) and DPPH (217.72 % of control). This also increased the beneficial aglycone form of isoflavones that includes daidzein (8.36-fold of control), glycitein (3.85-fold of control) and genistein (20.50-fold of control) but decreased the harmful β-glucoside form (3.65-fold) including daiazin (1.84-fold of control); glycitin (1.45-fold of control) and genistin (23.88-fold of control) over untreated dry seed. This may happen because of the conversion of conjugated β-glucoside isoflavones to their aglycone forms that have various health benefits. Maximum inhibition of NO production in RAW 264.7 cells was achieved by samples elicited for 2 h with 300 μg/mL concentration. This sample also confirmed the maximum anti-obesity activity treated against 3-T-3L1 cells. This study summarized that heat shock at 60 °C for 2 h increased polyphenols, antioxidants, and aglycon isoflavone in soybeans resulting in increased anti-inflammatory and anti-obesity activity.

## 1 Introduction

Soybean is rich in phytochemicals, nutrients [1], free radicals, and phenolic compounds [2,3] like cereals [4]. Phenolic content is highly associated with antioxidant activity [5]. The aglycon form isoflavones are the major flavonoids found in soybean seed, and they have diverse health effects [6]. Aglycones constitute a group of estrogenic substances responsible for several biological activities, acting as anti-cancer compounds, decreasing cholesterol levels, and reducing loss of bone mass [7]. Soybean-originated isoflavone has anti-inflammatory and anti-obesity activity like other plant sources [8,9]. Therefore, consumption of soybean seed isoflavones has several health-promoting properties such as lowering the rates of colon, prostate, and breast cancers; reducing blood cholesterol levels and risk of cardiovascular disease [10]; preventing osteoporosis and reducing post-menopausal symptoms [11,12].

Isoflavones in aglycone form are absorbed more rapidly with better utilization. β-Glucosides can be hydrolyzed to aglycones by a β-glucosidase enzyme released from intestinal microflora [13]. Limited availability of β-glucosidase enzyme in the intestine, time of food residence in the intestine, and types of diet and medicine intake may cause variation in isoflavone metabolism in the human body [14]. Therefore, the consumption of food with a high level of aglycone form isoflavone is desirable. Very small amounts of aglycones (1–3% of the total isoflavones) are present naturally in soybean seed. Some elicitation techniques such as soaking, fermentation [15], germination, dry-heat, wet-heat [16], coagulation, and protein precipitation can alter the distribution of the total isoflavone components in soybean seed. Physical and chemical stresses, UV light, low or high temperature, wounding, pathogens, and plant-microbe interactions as well as elicitor treatment also influence isoflavone content in soybeans [17]. Heat shock followed by soaking causes a decrease in Malonyl-CoA forms of isoflavone [18], while enzymatic hydrolysis with β-glycosidases increases aglycon forms [19].

It has been reported that hydrothermal heat shock elicitation causes the loss of considerable amounts (12–57 %) of isoflavone [20] and solubilizes high amounts of soy protein depending on heat shock duration and temperature [21]. This led us to look for an alternative technique that would prevent protein solubilization and isoflavone loss. We also aimed to increase the beneficial phytochemicals that have health impacts. Hence, the present study aimed to introduce a new elicitation technique that applied dry heat in moist seeds to increase the functional components and functionality. For this, seeds were subjected to dry heat in an oven following 6 h soaking in water. Subsequent analysis revealed that this technique increased both the functional components and functionality of soybean seeds.

## 2 Materials and methods

### 2.1 Cell culture

RAW264.7 macrophage cells were purchased from the American Type Culture Collection (Rockville, MD, USA) and nourished in Dulbecco's Modified Eagle's Medium (DMEM) added with 10 % fetal bovine serum, 1 % penicillin-streptomycin at 37 °C in a humidified incubator (MCO-18AIC, SANYO) with 5 % CO_2_ until the cells were grown to 80–90 % confluency. The cells were washed using Phosphate Buffer Saline (PBS) and harvested using a scraper according to Widowati et al. [22], Laksmitawati et al. [23].

Mouse fibroblast 3T3-L1 preadipocyte cells were used to check anti-obesity and obtained from the same authority. The cells were cultured in T-flask like RAW264.7 cells except supplementing 10 % newborn calf serum instead of fetal bovine serum. Each 48 h the media was changed. Cells were washed using Phosphate Buffer Saline (PBS) and harvested using Trypsin-EDTA (0.25 % Trypsin).

### 2.2 Chemicals

Tannic acid; Folin & Ciocalteu's phenol 2 N; 2,2′-azino-bis (3-ethylbenzothiazoline-6-sulfonic acid) (ABTS); 1,1-diphenyl-2-picrylhydrazyl (DPPH) were purchased from Sigma-Aldrich (Saint Louis, MO, USA). Daidzin, genistin, glycitin, daidzein, glycitein, and genistein standard chemicals were purchased from Chem Faces (Wuhan, Hubei 430,056, PRC). Dulbecco's Modified Eagles Medium (DMEM), Fetal Bovine Serum (FBS), Newborn Calf Serum, Penicillin/Streptomycin, Phosphate buffered saline (PBS, pH 7.4) and Trypsin-EDTA were purchased from Gibco, USA. MTT [3-(4,5-dimethylthiazol-2-yl)-2,5-diphenyltetrazoliumbromi-de], sulphanilamide, naphthyl-ethylenediamine dihydrochloride, lipopolysaccharide, insulin, dexamethasone and IBMX-3-isobutyl-1-methyl-xanthine isopropanol, Oil Red O staining dye and dimethyl sulfoxide (DMSO) were purchased from Duksan (Ansan, South Korea). RAW 264.7 and 3T3-L1 fibroblast cells were purchased from the American Type Culture Collection (Manassas, Virginia, USA).

### 2.3 Preparation of soybean seed samples from heat shock treatment

Soybean seeds were soaked in tap water for 6 h. After removing the water, the seeds were incubated at 50 °C, 60 °C, and 70 °C temperature for 1 h using the dry oven (EYELA NDO-700). Based on the maximum polyphenol content, isoflavone content, and antioxidant activity, 60 °C temperature was selected for further heat shock treatment. Then the seeds were incubated at 60 °C temperature for different durations (0.5 h, 1 h, 2 h, and 3 h). After cooling at room temperature, seeds were then dried overnight at 45 °C in an air dryer oven following Eshraq et al. [24]. Dried seed samples were ground with a laboratory grinder (KN 295 Knifetec) to make powder and sieved through a 100-mesh sieve. Prepared samples were preserved at 4 °C until chemical analysis. One-gram sample was added in 10 mL 70 % ethanol, shaken at 180 rpm for 3 h, centrifuged for 10 min at 4000 rpm and the supernatant was collected as sample extract. After syringe filtration (0.22 μm cut-off; PALL Corporation, USA), the extracted samples were preserved at 4 °C for further chemical analysis as mentioned by Lin and Lai [25].

### 2.4 Total polyphenol content determination

The total polyphenol content (TPC) of soybean seed extract was determined following the method described by Khatun and Mollah [26] with slight modifications. Briefly, 100 μL extracted sample was mixed with the same volume of Folin-Denis reagent and incubated for 3 min. Sodium carbonate (1 mL, 0.7 M) was added to this mixture and incubated for 1 h at room temperature to allow the chemical reaction. After 1 h incubation, absorbance was measured at 750 nm by using a microplate reader Molecular Device (Spectra Max ABS Plus). Untreated seed extracts were used as the control sample. To determine the total phenolic compounds, an equation obtained from the standard curve of tannic acid was used (0–500 μg/mL, *Y* = 0.0024*x* - 0.0071, *R*^2^ = 0.9993) where concentration was plotted on the X-axis and the corresponding absorbance on the Y-axis. The results were expressed as mg Tannic acid equivalents (TA eq.) per g of extract of seeds. All determinations were carried out in triplicate.

### 2.5 Antioxidant activity by free radical-scavenging activity using ABTS radical

Antioxidant activity was determined by the ABTS (2, 2′-azino-bis-3-ethylbenzthiazoline-6-sulphonic acid) free radical decolorization assay method developed by Re et al. [27]. The ABTS radical cation was regenerated by mixing ABTS stock solution (7 mM) with potassium persulfate (2.45 mM) (final concentration) and incubating for 12–16 h in the dark at room temperature until the reaction was completed and the absorbance was stable. The absorbance of the ABTS solution was equilibrated to 0.70 (±0.02) by diluting with water at room temperature, then 100 μL of this solution was mixed with 50 μL of the test sample and the absorbance was measured at 734 nm after 6 min. Untreated seed extracts were used as the control sample. All experiments were repeated thrice. The radical scavenging activities of samples were calculated by the following equation:

$$ABTS\;radical\;scavenging\;activity\left(\%\right)=\left(1-\frac{Sample\;absorbance}{Control\;absorbance}\right)\times 100$$

### 2.6 Antioxidant activity by free radical scavenging activity using DPPH radical

DPPH (1, 1-diphenyl-2-picrylhydrazyl) radical scavenging activity was determined using the method described by Khatun and Kim [28]. DPPH reagent was prepared by dissolving in 99.9 % ethanol. The reaction of DPPH and antioxidants yields a light-yellow color diphenyl picrylhydrazine. 50 μL of DPPH solution was incubated with 100 μL samples of different concentrations. Untreated seed extracts were used as a control. A control (Abs Control) containing methanol and DPPH solution was also realized. Blank control was treated with the solvent of the samples and DPPH reagent. All solutions were then incubated for 10 min at room temperature. Absorbance was measured at 517 nm. Radical scavenging capacity using free DPPH radical was evaluated by measuring the decrease of absorbance at 517 nm. The radical scavenging activities of samples were calculated as per the following equation:

$$DPPH\;Radical\;scavenging\;activity\left(\%\right)=\left(1-\frac{Sample\;absorbance}{Control\;absorbance}\right)\times 100$$

Then, curves were constructed by plotting the percentage of inhibition against concentration in μg/mL.

### 2.7 Quantification of isoflavones by high-performance liquid chromatography (HPLC)

The extracted soybean sample solution was filtered using a syringe filter (0.22 μm cut-off; PALL Corporation, USA) before high-performance liquid chromatography (HPLC) analysis. Standard daidzin, genistin, glycitin, daidzein, glycitein, and genistein were dissolved in 80 % methanol. The concentration of prepared authentic standards solutions ranged from 0.01 to 1 μg/mL for isoflavone analysis. For each standard, the mobile phase and its changing ratio are shown in Table 1. The extracted sample was dissolved in 1 mL of 10 % methanol and was ultra-sonicated for 10 min. From the methanol diluted sample, 10 μL of filtrates was directly injected into a high-performance liquid chromatography (HPLC) system. Each sample was analyzed in triplicate. The samples were analyzed by a C18 column (Shiseido Cap cell pack C18 MGⅡ, 3.0 mm × 250 mm, 5 μm). Phase separation was performed at 40 °C, the flow rate was maintained at 1 mL/min and the injection volume was 10 μL. The UV detection wavelength was measured at 260 nm wavelength using HPLC (High-Performance Liquid Chromatography, Shimadzu, Japan).

**Table 1:** HPLC analysis conditions for soybean seed isoflavones.

Time (min)	Solution A^a^ (%)	Solution B^b^ (%)
0	95	5
4	95	5
48	65	35
54	15	85
56	5	95
66	0	100
75	95	5

a Solution A was 0.1 % (v/v) acetic acid in water.

b Solution B was 0.1 % (v/v) acetic acid in acetonitrile.

### 2.8 Cell viability test

Cell viability was measured as described by García-Lafuente et al. [29]. This test used a colorimetric assay based on the principle that mitochondria of viable cells can reduce tetrazolium dye, MTT to its insoluble purple colored formazan. For this, RAW264.7 and 3-T-3L1 pre-adipocyte cells were seeded into 96 well plates at the density of 1 × 10^6^ cells/mL and incubated for 24 h. The cells were then treated with extracted samples and incubated for another 24 h for RAW264.7 cells and 48 h for 3-T-3L1 cells. Then the cells were treated with 10 μL of MTT (0.5 mg/mL in PBS) and incubated for another 3 h at 37 °C. After careful removal of the medium from the cell culture, dimethyl sulfoxide (DMSO) was added to dissolve formazan precipitates. Optical density was measured at 570 nm using a microplate reader Molecular Devices (Spectra Max ABS Plus).

After 24 h incubation, 10 μL of MTT (5 mg/mL in PBS) was added to each well, and cells were cultured for another 4 h at 28 °C. Formazan granules formed in viable cells were dissolved in 50 μL of DMSO. Absorbance was then measured at 540 nm using a microplate reader (Victor Multilabel Plate Reader, PerkinElmer, Waltham, MA, USA).

### 2.9 Anti-inflammatory activity estimation by NO product inhibition

Nitric Oxide (NO) product was determined using Griess reagent (1 % sulphanilamide, 0.1 % naphthyl ethylenediamine dihydrochloride, and 5 % phosphoric acid) following the method of García-Lafuente et al. [29]. RAW264.7 cells were seeded into 96-well plate at the density of 1 × 10^6^ cells/mL before 24 h of treatment application. Then, the medium was discarded and replaced with 0.2 mL of fresh medium without fetal bovine serum containing different sample concentrations. After 1 h incubation, LPS (Lipopolysaccharide; 1 μg/mL) stimulation was added and incubated at 37 °C for 24 h. The supernatant was collected from the cell culture medium and mixed with an equal volume of Griess reagent [equal volumes of 1 % (w/v) sulphanilamide in 5 % (v/v) phosphoric acid and 0.1 % (w/v) naphthyl ethylenediamine dihydrochloride] and incubated at room temperature for 10 min. Then the absorbance was measured at 540 nm using microplate reader Molecular Devices (Spectra Max ABS Plus). Each treatment was replicated thrice.

### 2.10 Anti-obesity activity estimation by adipocyte differentiation inhibition

For adipocyte differentiation, 3T3-L1 preadipocyte cells were seeded (1 × 10^5^ cells/mL) in 24-well plates. After 2 days of post-confluence (D_0_), 3T3-L1 preadipocytes were incubated with MDI (a mixture of 5 μg/mL insulin, 1 μM dexamethasone, and 0.5 mM 3-isobutyl-1-methyl-xanthine) in DMEM containing 10 % FBS along with sample extract for 48 h (D_2_). The cells were cultured for another 2 days (D_4_) in DMEM with 10 % FBS, insulin (5 μg/mL), and sample extract. Cells were incubated for another 4 days (D_8_) in DMEM with sample extract. By this time (D_8_) more than 90 % of cells became mature adipocytes with lipid-filled droplets and cells were fixed with 10 % formalin (pH 7.4) for 30 min. After washing with distilled water, cells were stained with diluted Oil Red O staining dye (Oil red O: water, 3:2), and the well plate was dried. The fat droplets were dissolved in 100 % isopropanol and the well plate was placed on a shaker at 350 rpm for 10 min. The relative fat accumulation was quantified by measuring the absorbance at 520 nm using a microplate reader Molecular Devices (Spectra Max ABS Plus). Inhibition ability was calculated following the formula of Yao et al. [30].

$$Inhibition\;ability\left(\%\right)=\left(1-\frac{OD\;Experimentalgroup}{OD\;Blank\;group}\right)\times 100$$

### 2.11 Statistical analysis

All assay data for continuous variables were conducted in triplicates. The values were analyzed using Microsoft Excel 2010, expressed as mean ± standard deviation (SD), and represented as graphs using SigmaPlot 12.5 software. All assay data were subjected to one-way analysis of variance (ANOVA) using PROG GLM in the SAS program [31]. Mean values were compared with Duncan's Multiple Range Test, *P* < 0.05.

## 3 Results

### 3.1 Effect of heat shock elicitation on polyphenol content and radical scavenging activity

Polyphenols have antioxidant and anti-inflammatory properties that can prevent major health concerns including cardiovascular disease, cancer, neurodegenerative disorders, obesity, etc. Heat shock elicitation increased the polyphenol content in soybeans (Table 2). Polyphenol content increased to 31.39, 36.49, and 32.18 mg/g extract upon heat shock at 50, 60, and 70 °C, respectively from control (23.48 mg/g). Thus, heat shock at 60 °C significantly (*P < 0.05*) increased polyphenol content (55.41 % over control). This result supports [32] who reported that at up to 60 °C polyphenol content increased significantly. Radical scavenging activity caused by Antioxidants also increased upon heat shock elicitation which was determined by ABTS and DPPH assays (Table 2). Radical scavenging activity by ABTS also increased significantly (*P < 0.05*) upon heat treatment. Radical scavenging activity increased to 43.23, 53.97, and 50.14 % at 50, 60, and 70 °C temperatures, respectively from control (33.92 %). For DPPH, radical scavenging activity increased to 10.16, 13.12, and 8.66 % at 50, 60, and 70 °C temperatures, respectively from control (7.80 %). Thus, maximum scavenging activity was obtained at 60 °C providing 59.11 and 68.21 % increase over control for ABTS and DPPH, respectively.

**Table 2:** Effect of heat shock elicitation on polyphenol content and antioxidant activity applied on soybean seed.

Heat shock	Polyphenol content (mg/g extract)	Radical scavenging activity (%) at 1000 ppm	
		ABTS	DPPH
Control	23.48 ± 1.14^c^	33.92 ± 1.04^d^	7.80 ± 0.36^b^
50 °C	31.39 ± 2.04^b^ (+33.67 %)	43.23 ± 2.24^c^ (+27.45 %)	10.16 ± 2.07^b^ (+30.26 %)
60 °C	36.49 ± 2.22^a^ (+55.41 %)	53.97 ± 1.98^a^ (+59.11 %)	13.12 ± 1.48^a^ (+68.21 %)
70 °C	32.18 ± 1.0^b^ (+37.05 %)	50.14 ± 3.20^b^ (+47.82 %)	8.66 ± 0.88^b^ (+11.03 %)

Means having different letters are significantly different (Duncan's Multiple Range Test.

*P* < 0.05). Percent reduction over control in parenthesis.

### 3.2 Effect of heat shock elicitation on isoflavone content

Isoflavones are not destroyed by heat rather converted to other forms. Isoflavone content in soybean seed was also elicited upon heat shock treatments (Table 3). A higher level of total β-glucoside content (1221.20 μg/g extract) was obtained from the untreated seed, which was decreased to 512.15, 553.31, and 533.90 μg/g extract upon heat shock at 50, 60 and 70 °C, respectively. Thus, the percent reduction was 58.06, 54.69, and 56.28 upon heat shock at 50, 60, and 70 °C, respectively compared to the control. On the other hand, total aglycone content increased exponentially in all the cases. Aglycon content increased to 115.08, 129.49, and 116.25 μg/g extract at 50, 60, and 70 °C, respectively from the control (12.50 μg/g extract). These ensured a 9.21-, 10.36-, and 9.30-fold increase of aglycon form isoflavone but decreased to 49.16, 44.65, and 47.30 % of total isoflavone content over control. Thus, the lowest β-glucosides but highest aglycones were obtained at 60 °C.

**Table 3:** Effect of heat shock elicitation on Isoflavone content applied on soybean seed.

Heat shock	Total β-glucosides (μg/g extract)	Total aglycone (μg/g extract)	Total Isoflavone (μg/g extract)
Control	1221.20^a^	12.50^c^	1233.70^a^
50 °C	512.15^d^ (−58.06 %)	115.08^b^ (9.21-fold)	627.22^d^ (−49.16 %)
60 °C	553.31^b^ (−54.69 %)	129.49^a^ (10.36-fold)	682.81^b^ (−44.65 %)
70 °C	533.90^c^ (−56.28 %)	116.25^b^ (9.31-fold)	650.14c (−47.30 %)

Means having different letters are significantly different (Duncan's Multiple Range.

Test, *P* < 0.05). Percent reduction and fold change over control in parenthesis.

### 3.3 Effect of heat shock duration on polyphenol content and radical scavenging activity

The duration of heat shock influences the elicitation of polyphenol content and radical scavenging activity (Table 4). Polyphenol content increased to 30.22, 31.87, 37.45, and 30.90 mg/g extract at heat shock duration of 30, 60, 120, and 180 min from control (23.31 mg/g extract). These durations increased 29.64, 36.72, 60.66, and 32.56 % polyphenol content over control, respectively. Thus, polyphenol was mostly elicited at 120 min of heat shock which was significantly (*P < 0.05*) different from others. A similar trend was observed for radical scavenging activity which was determined by ABTS and DPPH assays. Radical scavenging activity of ABTS and DPPH also increased significantly (*P < 0.05*) by heat shock duration (Table 4). For ABTS, heat shock for 120 min showed maximum (46.62 %) radical scavenging activity ensuring a 40.66 % increase over control. DPPH also showed maximum scavenging activity (23.67 %) at the same heat shock duration confirming an 18-fold increase over control. Thus, maximum scavenging activity was obtained upon heat shock for 120 min.

**Table 4:** Polyphenol content and antioxidant activity of heat shock elicited soybean seed sample at 60 °C temperature at different duration.

Heat shock duration	Polyphenol content (mg/g extract)	Radical scavenging activity (%) at 1000 ppm	
		ABTS	DPPH
Control	23.31 ± 0.21^c^	33.03 ± 1.16^e^	7.45 ± 0.94^d^
0.5 h	30.22 ± 2.27^b^ (+29.64 %)	36.62 ± 0.90^bc^ (+10.87 %)	15.95 ± 1.46^c^ (2.14-fold)
1 h	31.87 ± 0.32^b^ (+36.72 %)	40.46 ± 0.27^b^ (+22.49 %)	19.16 ± 0.43^b^ (2.57-fold)
2 h	37.45 ± 2.32^a^ (+60.66 %)	46.62 ± 1.25^a^ (+41.14 %)	23.67 ± 0.91^a^ (3.18-fold)
3 h	30.90 ± 1.72^b^ (+32.56 %)	38.32 ± 1.19^cd^ (+16.02 %)	16.93 ± 1.03^c^ (2.27-fold)

Means having different letters are significantly different (Duncan's Multiple Range Test.

*P* < 0.05). Percent reduction and fold change over control in parenthesis.

### 3.4 Effect of heat shock duration on β-glucoside isoflavone reduction

**Fig. 1:**
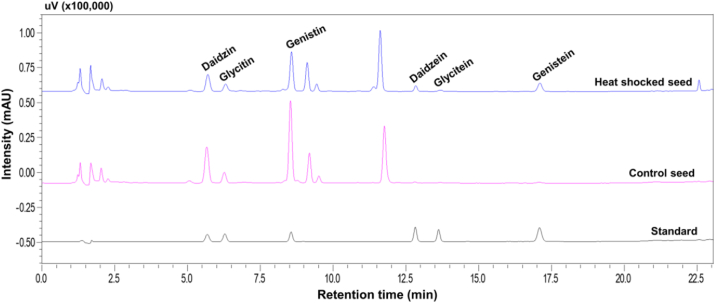
Detection of isoflavones by HPLC analysis from soybean seed samples heat shocked at 60 °C for 2 h compared with untreated dry seed and standard isoflavone chemicals.

Duration (min) of heat shock also influenced the β-glucoside content (Table 5; Fig. 1). In contrast to control, daidzin, glycitin, and genistin contents increased up to a certain level with the increase of heat shock duration, then decreased. Maximum daidzin was determined at 2 h heat shock which is statistically different (*P < 0.05*) from other forms of β-glucosides confirming 45.63 % reduction over control. A similar trend was observed for glycitin and genistin confirming 31.14 and 95.81 % reductions over control. Even, total β-glucosides were also decreased at 2 h duration which was 72.62 % less than the untreated control.

**Table 5:** Isoflavone (β-glucosides) profile (μg/g extract) of heat shock elicited soybean seed sample at 60 °C temperature at different duration.

Heat shock duration	β-glucosides (μg/g extract)			
	Daidzin	Glycitin	Genistin	Total
Control	412.73^a^	117.75^a^	690.72^a^	1221.20a
0.5 h	196.91^d^ (−52.29 %)	74.83^b^ (−36.45 %)	26.92^bc^ (−96.10 %)	298.65e (−75.54 %)
1 h	205.91^c^ (−50.11 %)	81.08^b^ (−31.14 %)	27.50^b^ (−96.02 %)	314.49c (−74.25 %)
2 h	224.41^b^ (−45.63 %)	81.08^b^ (−31.14 %)	28.92^b^ (−95.81 %)	334.40 b (−72.62 %)
3 h	207.41^c^ (−49.75 %)	74.83^b^ (−36.45 %)	25.42^c^ (−96.32 %)	307.65 d (−74.81 %)

Means having different letters are significantly different (Duncan's Multiple Range Test.

*P < 0.05*). Percent reduction and fold change over control in parenthesis.

### 3.5 Effect of heat shock duration on aglycone isoflavone induction

The content of aglycone form of isoflavone increased with the increase of heat shock duration (Table 6; Fig. 1). In control sample, aglycon isoflavone including daidzein, glycitein, and genistein was recorded as 6.08, 2.08, and 4.33 μg/g extract, respectively which increased to 50.83 μg/g extract for daidzein, 8.0 μg/g extract for glycitein and 88.75 μg/g extract for genistein after 2 h of heat treatment. This increase resulted in 8.36-, 3.85-, and 20.50-fold increase over control for daidzein, glycitein, and genistein, respectively. Contents of these isoflavones started to decrease after 2 h of heat treatment. Same phenomenon was observed for total aglycone content. Thus, maximum induction of aglycone isoflavones occurred at 2 h of heat shock treatment.

**Table 6:** Isoflavone (aglycone) profile (μg/g extract) of heat shock elicited soybean seed sample at 60 °C temperature at different duration.

Heat shock duration	Aglycone (μg/g extract)			
	Daidzein	Glycitein	Genistein	Total
Control	6.08^c^	2.08^d^	4.33^d^	12.50 d
0.5 h	42.33^b^ (6.96-fold)	7.67^b^ (3.69-fold)	75.58^b^ (17.45-fold)	125.58 b (10.05-fold)
1 h	40.83^b^ (6.72-fold)	7.00^b^ (3.37-fold)	71.58^c^ (16.53-fold)	119.41c (9.55-fold)
2 h	50.83^a^ (8.36-fold)	8.00^a^ (3.85-fold)	88.75^a^ (20.50-fold)	147.58a (11.81-fold)
3 h	41.75^b^ (6.87-fold)	5.92^c^ (2.85-fold)	74.16^b^ (17.13-fold)	121.83c (9.75-fold)

Means having different letters are significantly different (Duncan's Multiple Range Test.

*P < 0.05*). In parenthesis fold change over control.

### 3.6 Anti-inflammatory activity of aglycon isoflavone rich soybean sample induced by heat shock elicitation

**Fig. 2:**
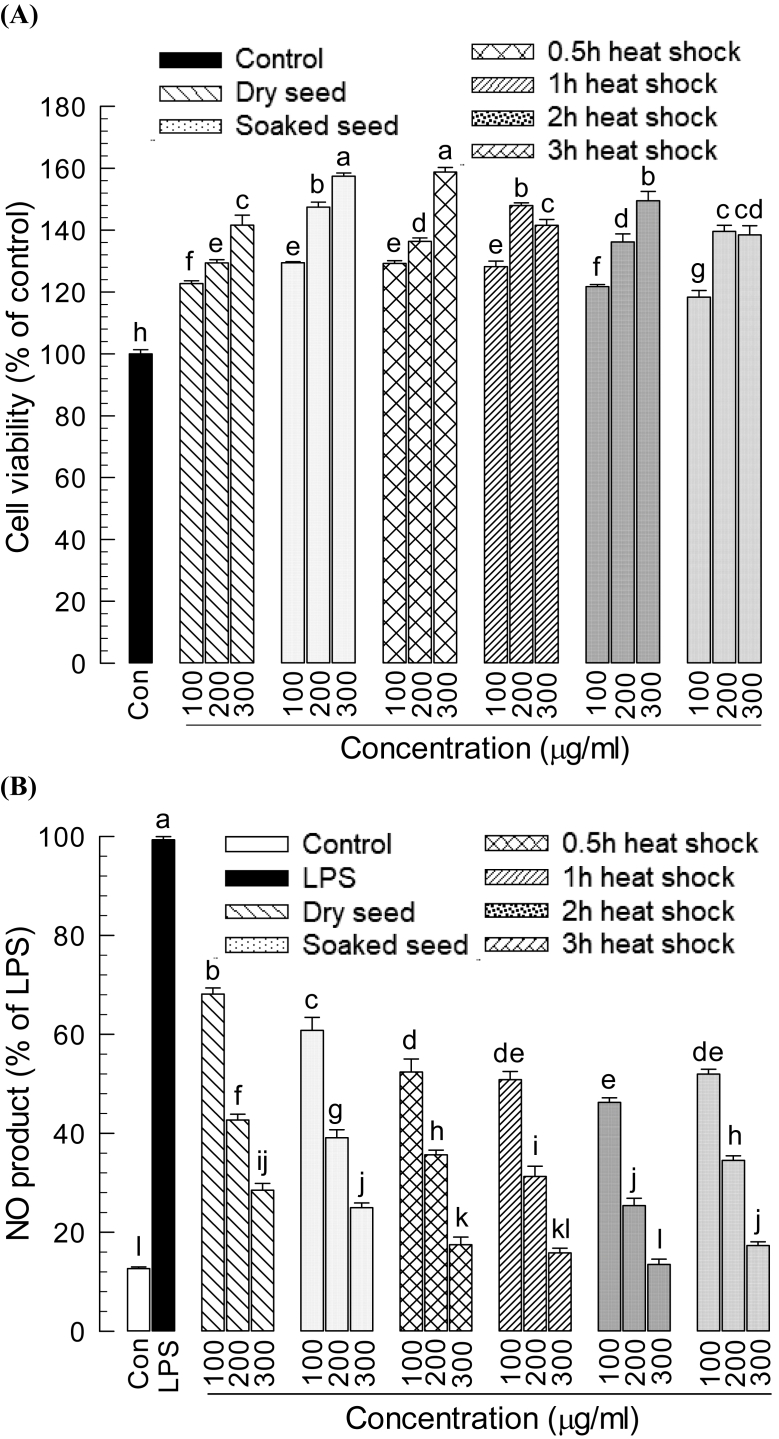
Effect of heat shock elicited soybean seed sample on RAW 264.7 cell. (A) Cell viability assay (B) Nitric oxide (NO) production in LPS stimulated RAW 264.7 cell. Cells were treated with samples of different concentrations (100, 200, 300 μg/mL) and duration (0.5 h, 1 h, 2 h and 3 h), and cell viability was analyzed by MTT assay at 540 nm absorbance. For NO production, cells were treated with different samples and incubated for 1 h then LPS (Lipopolysaccharide, 1 μg/mL) was added and incubated for 24 h. Control was not treated with LPS. After 24 h, NO product was determined at 540 nm using Griess reagent. Each treatment was replicated three times. Means having different letters are significantly different (Duncan's Multiple Range Test, *P* < 0.05).

Cytotoxicity of heat-elicited samples was evaluated by MTT assay against RAW 264.7 macrophage cells and found no toxic effect at any concentrations of 100, 200 or 300 μg/mL (Fig. 2A). Moreover, the duration of heat shock decreased the extent of toxicity and increased the rate of cell viability. Inhibition of Nitric oxide production indicated anti-inflammatory activity. To investigate the anti-inflammatory activity of the soybean sample, RAW 264.7 cells were treated with LPS and sample extract of different concentrations (100, 200, 300 μg/mL). The treated cells were cultured for 24 h and the accumulated NO production was measured. LPS (1 μg/mL) treatment significantly (*P* < 0.05) increased production of NO (Fig. 2B). However, elicited soybean samples significantly (*P* < 0.05) suppressed the NO production at different concentrations in a dose-dependent manner. Maximum inhibition ability was found for 2 h heat-elicited soybean sample even at very low concentration (100 μg/mL). For increased sample concentrations (200 and 300 μg/mL), the inhibition was statistically similar (for all durations except 1 h of heat shock (Fig. 2B). This result revealed that increased aglycon isoflavone resulting from heat shock elicitation inhibited NO product. All shocked elicited soybean samples had more potential for anti-inflammatory activity at 200 μg/mL dose.

### 3.7 Anti-obesity activity of aglycon isoflavone rich soybean sample induced by heat shock elicitation

**Fig. 3:**
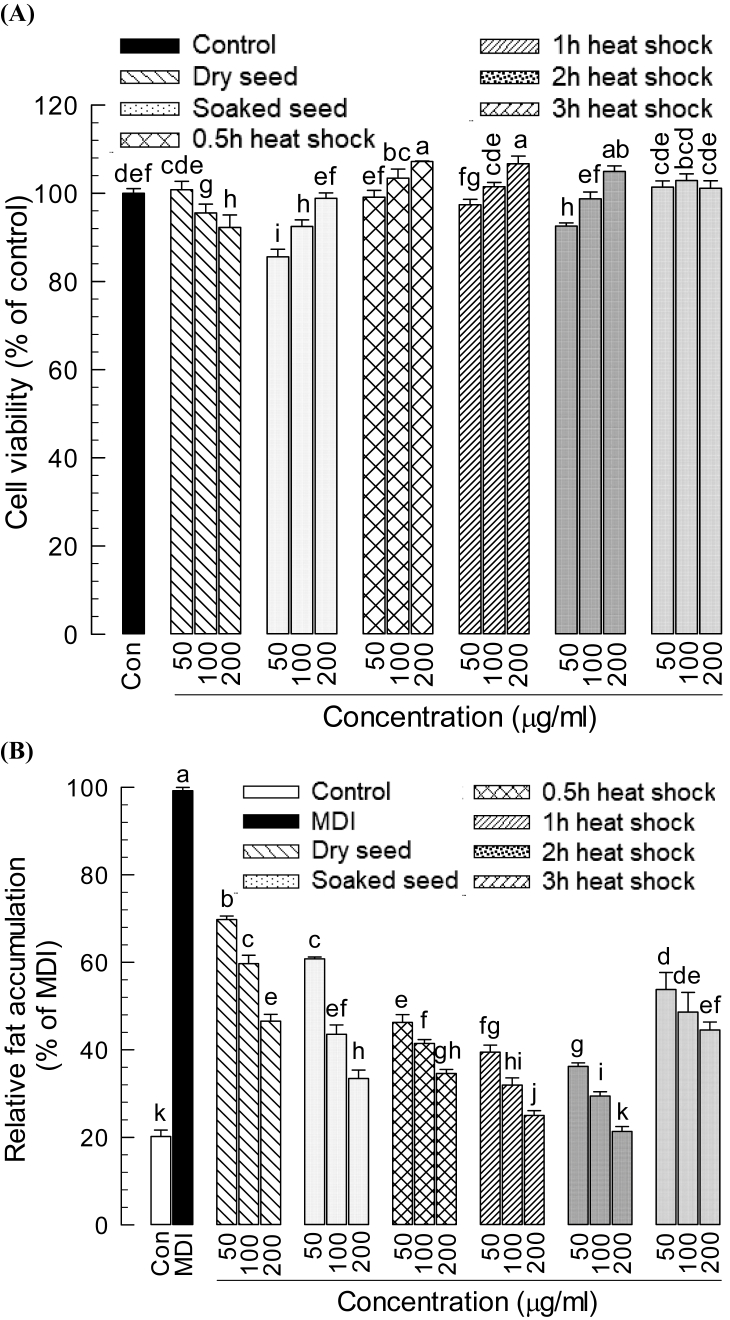
Effect of heat shock elicited soybean seed sample on 3-T-3L1 cell. (A) Cell viability assay (B) Adipogenic differentiation inhibition test. Cells were treated with samples of different concentrations (50, 100, and 200 μg/mL) and duration (0.5 h, 1 h, 2 h, and 3 h) and incubated for 48 h and cell viability was analyzed by MTT at 540 nm. Cells treated with different concentrations of the elicited sample were induced to differentiate into adipocytes by the MDI [0.5 mM 3-isobytyl-1-methylxanthine, 1 μM Dexamethasone, and 5 μg/mL insulin]. Following 8-day differentiation, adipocytes were fixed and stained with Oil Red O to visualize lipid droplets. Relative fat accumulation was quantified by measuring the absorbance at 520 nm. Each treatment was replicated three times. Means having different letters are significantly different (Duncan's Multiple Range Test, *P* < 0.05).

Cell viability was determined at the early adipocyte precursor (fibroblast) sage and adipogenesis was determined after differentiation as mature adipocytes (fat cells) using the Oil Red O stain. In this study, the cytotoxicity of heat shock elicited soybean sample was determined by testing the viability of 3-T-3L1 fibroblast cells using MTT. All samples showed cell viability levels above 80 % whereas heat elicited samples confirmed more than 90 % cell viability (Fig. 3A). Therefore, all samples were non-toxic to preadipocytes after 48 h of incubation with different concentrations (50, 100 and 200 μg/mL) of soybean heat elicited sample compared to the control.

Adipogenesis was determined by the accumulation of lipid droplets staining with Oil Red O stain. During preadipocyte differentiation process, the adipocytes were treated with various concentrations (50, 100 and 200 μg/mL) of elicited soybean sample. All heat-elicited samples significantly (*P* < 0.05) suppressed the fat accumulation at any concentration of 50, 100 and 200 μg/mL in a dose-dependent manner except that of the 3 h elicited sample. All doses of 2 h heat-shocked samples allowed minimum fat accumulation compared to the respective dose of other samples and the highest dose (200 μg/mL) performed like the control (Fig. 3B). The fat accumulation inhibition ability of other samples was significant (*P* < 0.05) compared to the unelicited dry seed sample. Thus, a 2 h heat shock increased the anti-obesity properties of soybeans by inhibiting fat accumulation potentially.

## 4 Discussion

The present study revealed that heat shock at 60 °C for 2 h increased the polyphenol content as well as antioxidant activity in soybeans. A previous study [33] reported that steaming increased the polyphenol content and antioxidant activity in bean milk. Another study [34,35] described the interrelations of polyphenol content and antioxidant activity. Increased antioxidant activity of phenolics might come from their benzene rings which have hydrophobic properties to donate hydrogen from the hydroxyl group and can chelate metals. The resulting hydroxycinnamic compounds have a relationship with antioxidant activity [36].

Heat shock also induced the readily available aglycone form of isoflavone to have high biological activity but decrease the harmful β-glucoside form of isoflavone. A previous study [8] supports these findings reporting that in heat-shocked moist seed, β-glycosidase enzyme hydrolyses β-glucosides to aglycon. Moreover, malonyl form β-glucosides are heat sensitive and decreased by heat shock [18]. Heat treatment of soybean at 55 °C for 5 h also enhanced the conversion of conjugated isoflavones to their aglycone forms due to the action of their endogenous β-glucosidases enzyme [37]. Heat treatment of soybean seeds at 60 °C for 1 h also increased the isoflavone content compared to non-heated seeds [38]. These explained how aglycon increased in soybean seed upon heat treatment. Heat shock at 60 °C for 2 h also increased the functionality of soybean seeds providing enhanced anti-inflammatory and anti-obesity activity over the control. This might be explained by the increased polyphenols, antioxidants, and aglycone isoflavones that have synergistic effects against obesity and inflammation. A previous study described that increased aglycon content promotes anti-inflammatory activity by inhibiting NO production and anti-obesity activity by inhibiting fat accumulation [8,9]. The steamed soybean also increased anti-obesity activity in rat systems [39].

Therefore, heat treatment at 60 °C for 2 h was found very crucial to increase the functional component as well as functionality in soybeans. For this, polyphenols, antioxidants, isoflavones, anti-obesity, and anti-inflammatory activity were analyzed with the sample extracts that were treated at 50, 60, and 70 °C but samples treated at 60 °C induced phytochemicals mostly. For the duration, the same analysis was conducted with the samples subjected to heat shock at 60 °C for 0.5 h, 1 h, 2 h, and 3 h. Among these durations, maximum induction of the components was observed for the samples heat shocked for 2 h. Further research will facilitate the optimization of the heat shock parameters and find out the conversion mechanisms.

## 5 Conclusion

Heat shock on moist soybean seed for 2 h at 60 °C exponentially elicited beneficial functional components like polyphenol, antioxidants, and aglycon isoflavone but decreased harmful β-glucosides compared to control. This also increased the anti-obesity and anti-inflammatory properties of soybean seeds. This easy and innovative technique will promote the food value as well as the market demand for soybeans. However, for other grains, this elicitation can be applied. Further research on the optimization of heat shock parameters and elucidation of conversion mechanisms will help achieve the goal.

## Funding statement

This research did not receive any specific grant from funding agencies in the public, commercial, or not-for-profit sectors.

## Data availability statement

Data included in article/supplementary material/referenced in article.

## CRediT authorship contribution statement

**Soyema Khatun:** Writing – review & editing, Writing – original draft, Visualization, Methodology, Formal analysis, Data curation, Conceptualization. **Taewan Kim:** Writing – review & editing, Validation, Supervision, Software, Resources, Project administration, Funding acquisition. **Md Mahi Imam Mollah:** Writing – review & editing, Software, Formal analysis, Data curation.

## Declaration of competing interest

The authors declare having no potential conflicts of interest relevant to this study to disclose.
